# Developing a logic model for communication-based interventions on antimicrobial resistance (AMR)

**DOI:** 10.1371/journal.pgph.0002965

**Published:** 2024-06-13

**Authors:** Jennika Virhia, Emma Laurie, Tiziana Lembo, Jeremiah Seni, Roxana Pollack, Alicia Davis, Siana Mapunjo, Stephen E. Mshana, Blandina T. Mmbaga, Shona Hilton

**Affiliations:** 1 MRC/CSO School of Social & Public Health Sciences Unit/School of Health and Wellbeing, University of Glasgow, Glasgow, United Kingdom; 2 School of Geographical and Earth Sciences, University of Glasgow, Glasgow, United Kingdom; 3 School of Biodiversity, One Health & Veterinary Medicine, University of Glasgow, Glasgow, United Kingdom; 4 Department of Microbiology and Immunology, Weill-Bugando School of Medicine, Catholic University of Health and Allied Sciences, Bugando Medical Centre, Mwanza, Tanzania; 5 School of Social and Political Sciences/School of Health and Wellbeing, University of Glasgow, Glasgow, United Kingdom; 6 National Institute for Medical Research and Ministry of Health, Dodoma, Tanzania; 7 Kilimanjaro Clinical Research Institute, Kilimanjaro Christian Medical Centre, Moshi, Tanzania; St John's National Academy of Health Sciences, INDIA

## Abstract

The importance of communication in enhancing people’s awareness and understanding of antimicrobial resistance (AMR) is consistently recognised in global and national action plans (NAPs). Despite this, there have been relatively few national AMR communication campaigns which use a structured approach to take account of the local context, encompass co-design with the target audience and use a logic model to help inform its design, implementation and evaluation. Designing a logic model for communication-based interventions can help map out the planning, resources, messaging, assumptions and intended outcomes of the campaign to maximise its impact, ensure it is fit for context and minimise any unintended consequences on individuals and society. Building on an AMR research project in Tanzania, Supporting the National Action Plan for AMR (SNAP-AMR), we co-designed the SNAP-AMR Logic Model with key stakeholders to implement AMR communication campaigns and related legacy materials to be employed in support of the Tanzanian NAP, but with broader relevance to a range of contexts. In developing the SNAP-AMR Logic Model, we reviewed relevant communication theories to create and target messages, and we considered behavioural change theories. We defined all key elements of the SNAP-AMR Logic Model as follows: (1) resources (inputs) required to enable the design and implementation of campaigns, e.g. funding, expertise and facilities; (2) activities, e.g. co-design of workshops (to define audience, content, messages and means of delivery), developing and testing of materials and data collection for evaluation purposes; (3) immediate deliverables (outputs) such as the production of legacy materials and toolkits; and (4) changes (outcomes) the campaigns aim to deliver, e.g. in social cognition and behaviours. The SNAP-AMR Logic Model efficiently captures all the elements required to design, deliver and evaluate AMR communication-based interventions, hence providing government and advocacy stakeholders with a valuable tool to implement their own campaigns. The model has potential to be rolled out to other countries with similar AMR socio-cultural, epidemiological and economic contexts.

## Introduction

Effective communication plays a pivotal role in public health and is a fundamental component of national strategies aimed at addressing the urgent global health challenge presented by antimicrobial resistance (AMR). By 2050, AMR has been predicted to be responsible for around 10 million global deaths each year, with the greatest burden of mortality being in low-resource settings (LRSs) [1]. For example, recent estimates suggest that, in sub-Saharan Africa, 75 of 100,000 deaths were directly associated with AMR in 2019 [2]. With such dire predictions of AMR’s future growth across the globe and with LRSs being more vulnerable to its spread and impacts, AMR will further widen global health inequalities, threatening every aspect of medicine from the infectious to the non-infectious diseases. Urgent action is therefore needed to tackle this major health crisis. To combat AMR, the World Health Organization (WHO) developed a Global Action Plan (GAP) for AMR in 2015 [3], urging all member countries to develop National Action Plans (NAPs). Common to all NAPs are strategic objectives for tackling AMR. The first strategic objective highlights the need for improving awareness and understanding of AMR among policy makers, professionals and the public ‘through effective communication, education and training’ [3]. Designing effective health communication campaigns is, therefore, important in mobilising coordinated support and effort across sectors. Developing an understanding of the issue first helps guide how the problem is defined, who is accountable, who will be impacted and, crucially, how the solutions are framed [4]. However, understanding and communicating about AMR is challenging given the multi-faceted nature of this problem. As such, there is no clear ‘silver bullet’ solution. The drivers of AMR are interconnected ranging from the biomedical to the socio-political and economic, with multiple systems affected. This makes identifying the leverage points and communicating the ‘sources’ or causes of AMR difficult and context dependent. Communication is further complicated by the invisibility of AMR. Despite evidence of multiple impacts, such as increased incidence of infection, prolonged treatment times and treatment failure [5], these are hard for the general public to attribute directly to AMR. The multitude of terms used to describe the threat from AMR complicate communication further [6, 7]. These challenges highlight that awareness campaigns need to be informed by evidence on what might be best predicted to work when communicating such complex issues and, indeed, what is possible and appropriate to the target audience.

Given the multifaceted and complex challenges around communicating AMR we discuss above, it is unsurprising that few AMR campaigns have been developed, especially in LRSs. In a review by Huttner et al., (2019) [8], 60 AMR campaigns were identified overall. Of these, only three (5%) were from the African Region [8]. The rest were from the European region (58%), the Americas (15%), South-East Asia (10%), Western Pacific (8%) and Eastern Mediterranean region (3%) [8]. The authors of the review examined the characteristics of these campaigns and found that not all of them were scientifically sound. For example, they lacked a thorough evaluation and the multifaceted nature of the campaigns rendered it difficult to make any inferences about their overall effectiveness. The majority of the campaigns in the study targeted health practitioners and the general public simultaneously and utilised a multi-pronged approach consisting of education and communication material, online platforms, televisions and radio. Other campaigns were comprised of public relation activities, press conferences, educational meetings for prescribers and active promotion of treatment guidelines. Of note, only 13 out of 60 (22%) of the campaigns considered a One Health approach in their design. Many of those living in LRSs who are most affected by AMR live in close proximity to their animals within their shared environments which could expose them to animal and environmental transmission of antibiotic resistant bacteria. AMR transmission can spread through the food chain which are reasons for concern beyond LRSs. Therefore, framing AMR as an issue that goes beyond human health is essential in expanding its defined impacts to incorporate issues around sustainability and the significant risks that AMR transmission pose to global food security, animal welfare and the natural environment.

Another issue identified in earlier campaigns is that much of the messaging used focussed on individual (mis)use of antibiotics, e.g. ‘If we use antibiotics incorrectly we will lose them/they will become ineffective’ [8]. Using negatively framed messages without concrete action points places a disproportionate responsibility on individuals to make optimal health decisions. This type of campaign message lacks the necessary actionable guidance on small scale preventative measures, whilst also overlooking the socio-political, cultural and economic environments within which individuals live and operate and that are likely to restrict their choices and agency [9–12]. The situated realities of these environments animate individuals’ relations with antimicrobials and can inhibit optimal antimicrobial use. Furthermore, these individualistic responsibility frames that may be appropriate in High Resource Settings (HRSs) risk becoming ‘blame narratives’ in LRSs where access to health care, diagnostics and clean water are limited to large sections of the population [13]. While there is a need to address the structural shortcomings in health infrastructure, and to avoid placing all responsibility on individuals, communication campaigns can be an effective tool to equip individuals with small-scale positive daily actions to protect themselves (such as, for example, completing the full course of antibiotics) and improving knowledge and awareness of AMR (which can then be leveraged by those advocating for equitable policy change).

Logic models have been recognised for the range of benefits they provide when designing communication campaigns, including: developing a common language; participatory decision making; and providing a reporting framework and overall improvement to campaign design, planning and management [14]. Logic models typically comprise clearly defined elements. The Centers for Disease Control and Prevention (CDC) describes these as: inputs (the resources needed to implement activities), processes or activities (what we do with the resources in order to produce particular results), outputs (the immediate deliverables or tangibles that result from given inputs and activities) and outcomes (the changes in people or conditions arising from the activities or outputs—these might be short-, intermediate- or long-term). These elements are typically visually displayed within a framework that shows the connections or flow between them. Logic models are increasingly used in HRSs in health communication programmes to assess the links between interventions (such as raising awareness) and any changes in health outcomes (such as changed behaviour around risky health practices) [15]. Designing a logic model can help recognise the complexity of delivering health interventions such as health communication campaigns. They can, therefore, ensure that all components of an effective campaign are captured in the planning and implementation process.

Despite the benefits of developing logic models tailored to health communication interventions, there are relatively few studies that have used these tools for communicating AMR [16], especially in the African context. More broadly, to date, AMR policy interventions in general have been characterised as ad hoc rather than incorporating an explicit process that considers the determinants of the problem, relevant theory and available empirical evidence [17, 18]. This has resulted in poorly implemented AMR policy that aims to change knowledge, attitudes, beliefs and practices around AMR, without clearly articulating how these interventions will bring about this change [17]. This approach also fails to take into account the wider context which may lead to ineffective or sub-optimal interventions.

Logic models can help improve the design of AMR communication-based interventions by explicitly describing how and why an intervention is expected to work. This is helpful in AMR research where there is a tendency to try and re-invent the wheel rather than build on existing evidence from behavioural and implementation science [17]. To address this gap, in this paper, we describe the development of a logic model related to AMR communication campaigns in the Tanzanian context, including in communities (both urban and rural) and in hospital settings. The logic model was built as part of a research project, Supporting the National Action Plan for Antimicrobial Resistance in Tanzania (SNAP-AMR). This interdisciplinary project was designed around the strategic objectives of the Tanzanian NAPs 2017–2022 [19] and 2023–2028 [20], which, in line with the GAP, emphasise the importance of increasing AMR awareness through effective communication. The Tanzanian NAPs provide an overarching framework on AMR communication, and while AMR communication campaigns have been implemented (e.g. via participation in World Antimicrobial Awareness Week (WAAW)), the results (outputs, outcomes and impacts) of these campaigns were not objectively assessed and remain to be further explored based on existing NAP-AMR implementation activities [21]. The campaigns conducted within the SNAP-AMR project therefore highlight the effectiveness of using the SNAP-AMR Logic Model to map out every stage of the campaign to ensure impacts are well captured and attributable to campaign activities. We envisage that the logic model will provide a framework that can be adapted for the development of campaigns tackling a range of health communication issues across low-, middle- and high-income countries.

## Methods

### Process of designing the SNAP-AMR logic model

#### Step 1: Guiding principles and approach

**Fig 1:**
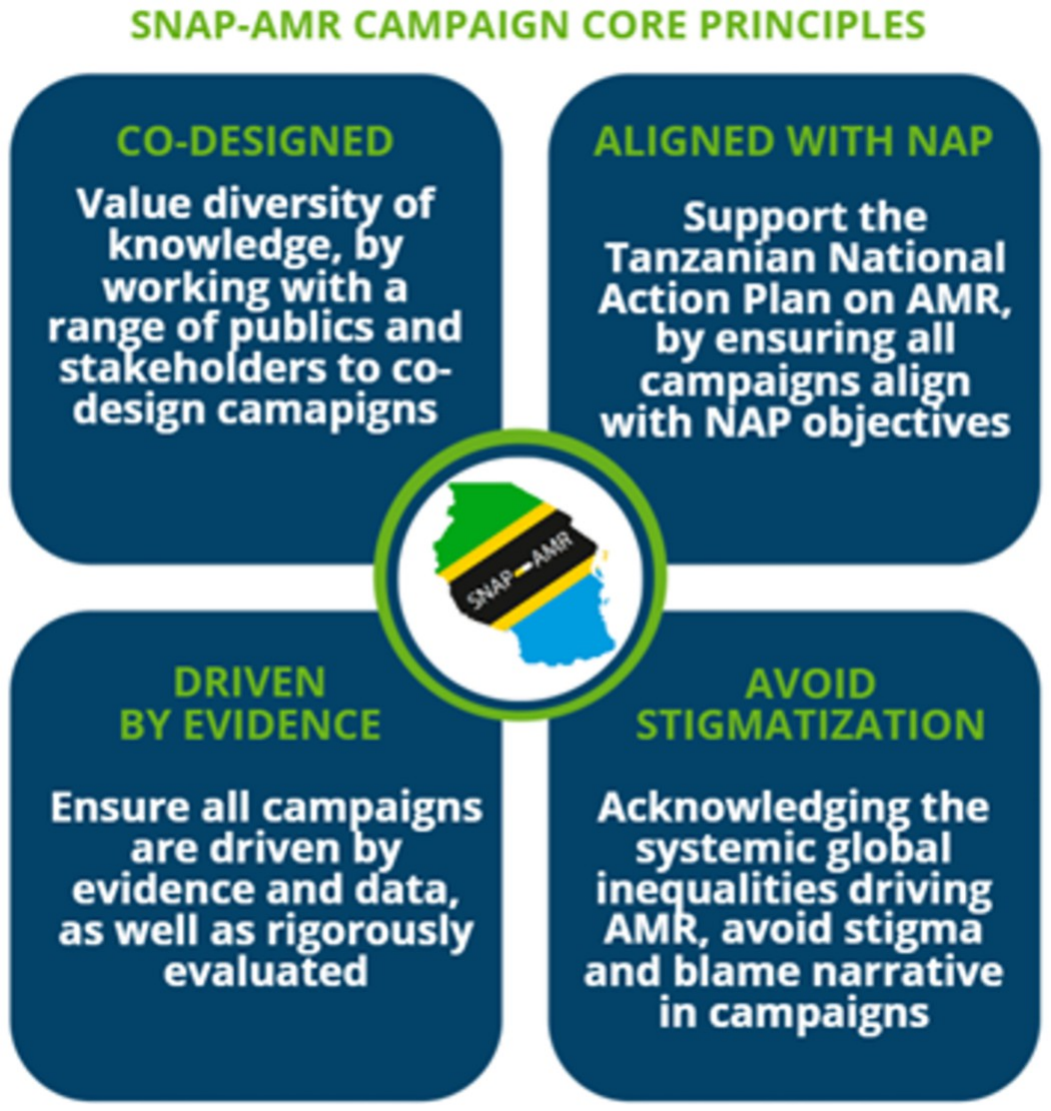
Guiding principles of the project Supporting the National Action Plan for Antimicrobial Resistance (SNAP-AMR) in Tanzania. Source: SNAP-AMR AMR Campaign Toolkit: A practical toolkit for delivering antimicrobial awareness raising campaigns (forthcoming).

The development of the SNAP-AMR Logic Model, and associated campaigns, was underpinned by the project’s guiding principles, shown in Fig 1.

The guiding principles were agreed upon by the SNAP-AMR research team and wider project advisers at the outset of the project. These principles were used to ensure that an ethics of care and responsibility sat at the heart of the conduct of the research team, as well as in designing and implementing the campaigns. The principles were informed by engagement with ongoing debates on conducting ethical and equitable global health research, including literature on decolonising global health (see: [22, 23]). Recognising that global health inequalities contribute to AMR and will worsen as the global AMR burden increases, we focused on campaigns aimed at empowering through education and information, and used language that avoided stigmatisation and blaming narratives (see Laurie et al., forthcoming). Core principles of co-design, alignment with the Tanzanian National Action Plan for AMR and a commitment to be driven by data also guided our approach. Care was taken to ensure that all core principles were imbued in the process of designing the SNAP-AMR Logic Model, and ensured the campaigns were collaborative, evidence-informed, fit for context and took a holistic approach to AMR communication. Additional information regarding the ethical, cultural and scientific considerations specific to inclusivity in global research is included in the Supporting Information (S1 Checklist).

#### Step 2: Establishing appropriate theoretical framing

The initial phase of development of the SNAP-AMR Logic Model involved a review of reviews [16] that generated the best theoretical evidence in the research literature to clearly define the model’s components and linkages. The review identified several theories that may contribute to effectiveness of campaigns, some of which are explained in Box 1. Elements were used from these theories to develop the SNAP-AMR Logic Model which underpinned the planning, development and implementation of the communication campaigns. For example, communication theories were used to inform how best to create and target messages, while behavioural change theories were drawn upon to understand how different people might respond to such messages [16].

**Box 1. Theories and frameworks underpinning the development of the SNAP-AMR Logic Model.**

*Social marketing theory* [24–27]—A collection of theories that considers health as an important market. Health professionals, therefore, must know how to market key concepts and design programmes to promote health products and change health behaviours [28]. Social marketing focuses on how socially valuable information can be promoted in order to persuade people to accept ideas and attitudes, perform healthy behaviours, refer to health facilities and receive health products. The target audience is identified based on their information need. Once this is done, information is packaged and distributed in a manner that will be easily accessible to the intended audience.

*Cultivation theory* [29]–A sociological and communication framework which holds that regular and long-term exposure to media influences how consumers of media perceive the world and behave in real life [30]. Cultivation theory posits that, over time, if a person hears, reads or sees a repeat message, this is more likely to resonate. This theory also predicts that people accumulate knowledge and abilities that serve as building blocks for subsequent cognitive development. This process is largely the gradual development of knowledge and skills that improve over time.

*Framing* [31]—How an issue is ‘framed’, i.e. explained and presented through specific themes and angles, is key to clearly defining a problem, who is accountable, who will be impacted and, importantly, the solutions. Framing can also influence how messages are received by an audience. It can mean that people are more likely to understand, engage with and support action on an issue or, on the contrary, oppose it.

*Diffusion of innovation* [32]—This theory suggests that over time novel ideas and behaviours spread through members of a social system via communication channels with peer-supporters acting as early adopters or innovators.

While behaviour change and communication theories are useful for explaining individual cognitive processes resulting from exposure to information, they are often critiqued for their individualistic bias [33–34] and neglect the real-world constraints that might operate in the individual’s environment, particularly in LRSs [35]. In line with the guiding principles (Fig 1), the SNAP-AMR project also adopted a broad social-ecological framework for the design of its campaigns. Such an approach is vital for recognising the complex interplay between individuals, communities, health systems and societal factors driving AMR. A social-ecological framework acknowledges that individual behaviours shape and are shaped by broader social, economic, political and environmental factors [36–38], all of which contribute to AMR. This approach ensured that SNAP-AMR campaigns would be developed to take account of the wider contextual determinants impacting health, to better support change and facilitate change in health behaviour.

#### Step 3: Refinement via co-design

In line with SNAP-AMR core principle of co-design, we recognise and value the diverse knowledges held by different stakeholders within and external to the project. The SNAP-AMR Logic Model and associated campaigns were therefore co-designed by researchers, animal and human health providers, community members, community leaders and members of the Tanzanian Ministry of Health and Ministry of Livestock and Fisheries.

The initial model was introduced during project development in Moshi, Tanzania, in November 2017 and refined at a workshop with the SNAP-AMR team in Mwanza, Tanzania, in December 2018. Further refinement took place during AMR expert workshops held the following year in Arusha, Tanzania, in December 2019, with a final evaluation meeting in Glasgow, Scotland, in March 2023. The workshops in Tanzania examined the constructs in the SNAP-AMR Logic Model for communicating AMR in the Tanzanian context specifically, with experts interrogating the model from their unique perspectives to further develop it. The reflections and lessons learnt from these stakeholders helped refine model’s inputs, activities, outputs and outcomes in an iterative process. Specifically, this information helped the research team to revisit the information presented in the model to ensure its accuracy and completeness, so that it could be used to tell the story of how an AMR awareness communication campaign can be effectively mobilised in Tanzania. These interactions also enabled the researchers to test their assumptions related to the model components and the linkages among them. This iterative and repetitive process was essential, as logic model development guides stress the importance of reviewing and updating the logic model to maintain its fidelity as changes occur (c.f. [39–41]).

Discussions with diverse stakeholders provided vital insight into what each component of the SNAP-AMR Logic Model should contain for effective implementation of AMR communication campaigns in northern Tanzania. The campaigns were designed to specifically support the Tanzanian NAP but also had broader relevance to, and applicability in, a range of contexts. The team tested and implemented the SNAP-AMR Logic Model across three settings: two neonatal wards located in two zonal hospitals in northern Tanzania; amongst the general public using public vehicles on local transport routes to hospitals; and in a rural livestock dependent community. The details of these campaigns are discussed in the results section.

All campaign participants were recruited and data were collected between 1st April– 31st May 2019 and 1st September 2020–28th February 2021. All participants were explained study objectives and written and/or verbal informed consent was obtained. Verbal consent was utilised instead of written consent with participants who were unable to write.

#### Ethics statement

The study received approval from the Kilimanjaro Christian Medical University College Ethics Review committee with certificate n. 2408 and the Catholic University Health and Allied Sciences committee with certificate n. CREC/318/2018; National Institute for Medical Research (NIMR), Tanzania, with Reference Number NIMR/HQ/R.8a/Vol. IX/3017; Tanzanian Commission for Science and Technology; and College of Medical Veterinary and Life Sciences ethics committee at the University of Glasgow (project application number 200180046). Permission for research in communities was obtained from relevant local and district authorities. All participants were explained study objectives and written and/or verbal informed consent was obtained. Verbal consent was utilised instead of written consent with participants who were unable to write. Verbal consent was achieved via the participant having the statement of consent read to them, followed by signatures from the person taking the consent and a witness. Informed consent forms and participant information sheets were approved by all ethics panels.

## Results: The SNAP-AMR logic model

### Describing the SNAP-AMR logic model

**Fig 2:**
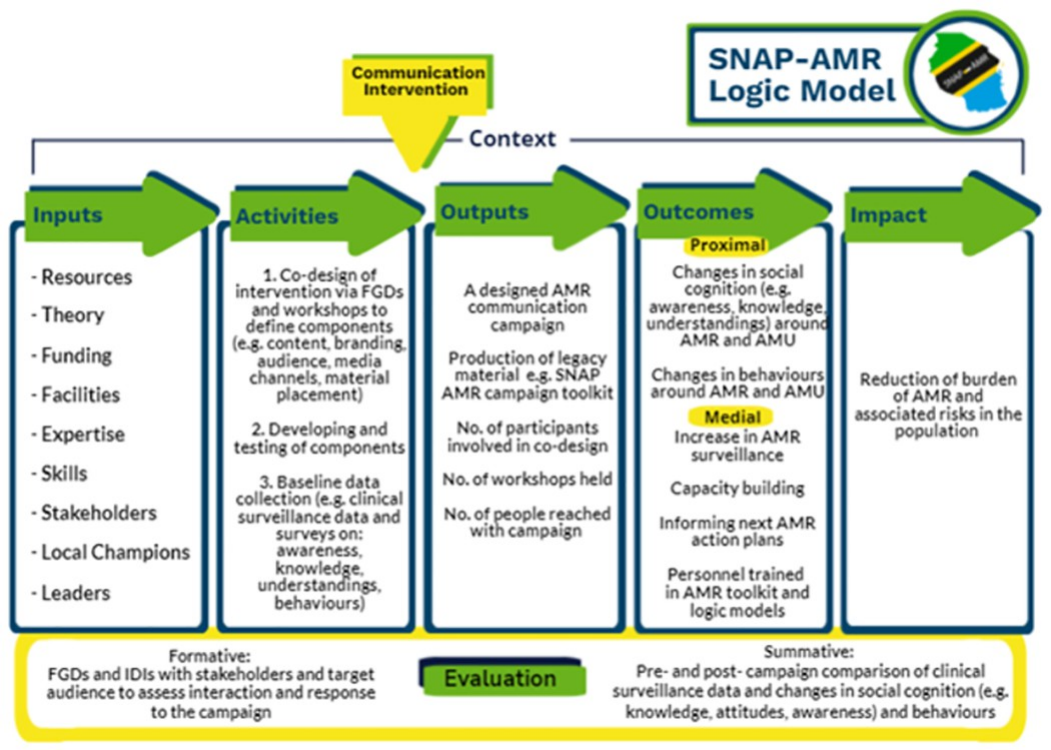
The SNAP-AMR (Supporting the National Action Plan for Antimicrobial Resistance in Tanzania) Logic Model. Abbreviations: AMR—Antimicrobial Resistance; AMU—Antimicrobial Use; FGD—Focus Group Discussion; IDI—In-depth Interview; SNAP-AMR—Supporting the National Action Plan for Antimicrobial Resistance.

The final SNAP-AMR Logic Model can be seen in Fig 2. The overall format of the model was informed by CDC guidance, and the component parts resulted from several iterations and discussions with stakeholders described above. Logic Models are traditionally presented as a staged, diagrammatic format such as in Fig 2, which we wanted to align with.

Regarding the model, it was agreed that the ultimate goal of effective communication campaigns should be to reduce AMR burden and associated risks (impact). While we recognise that achieving such impact will require behaviour change, effective communication campaigns construct the required foundation to achieve this, and also align with the priority of the NAP in Tanzania and, thus, the guiding principles detailed in Fig 1. We then identified the: (1) resources (inputs) required, e.g., funding, expertise, facilities and local champions; (2) activities that should be conducted to define the audience, content, messages and means of delivery to develop and test the materials, and to evaluate the campaigns; (3) immediate deliverables (outputs), e.g., the production of legacy materials and toolkits; and (4) changes (outcomes) the campaigns aim to deliver, e.g., in social cognition and behaviours. At all stages, an understanding of the context was vital to ensure campaigns were place-based and tailored to the local context. We utilised reflective, probing questions at each stage to help identify the configuration of each component of the SNAP-AMR Logic Model. To improve the utility of the model we have included Table 1 which provides additional points, examples and probing questions that potential users of the logic model may want to consider when designing an effective communication campaign.

**Table 1:** Description of components, associated probing questions and related examples of the SNAP-AMR (Supporting the National Action Plan for Antimicrobial Resistance in Tanzania) Logic Model. The full list of probing questions can be found in the SNAP-AMR AMR Campaign Toolkit: A practical toolkit for delivering antimicrobial awareness raising campaigns (forthcoming).

Components of the SNAP-AMR Logic Model	Probing questions	Examples from SNAP-AMR campaigns
Inputs	What resources are needed in order to deliver the activities of the campaign?	Human and financial resources Appropriate theoretical framework
Activities	Using the inputs available, what activities will be undertaken in order to produce the desired outputs of the campaign?	Co-design workshops to define elements of the campaign including target audience, messages and means of delivery Developing and testing components Baseline data collection (surveillance data and surveys/interviews on knowledge, awareness, understandings and behaviours)
Outputs	What immediate deliverables or tangibles can be expected based on campaign activities?	A designed AMR communication campaign Production of legacy material e.g. toolkit No. of people involved in co-design No. of people reached with campaign No. of workshops/training sessions held No. of people completing the training
Outcomes	What are the expected changes that can be influenced by the campaign (in the short, medium and long term)?	Proximal Changes in social cognition e.g. awareness, understanding, knowledge, attitudes, intention, motivation Changes in behaviour
		Medial Increase in AMR surveillance Capacity building Informing next action plans People trained in AMR toolkit and logic models
Impact	What long term or distal desired changes does the campaign contribute to?	Reduction of burden of AMR and associated risks in the population
Evaluation	How will the campaign be evaluated to test feasibility, appropriateness and acceptability within the target audience?	Focus group discussions and in-depth interviews with stakeholders and representatives of target audience to assess how participants interact and respond to the campaign, and to avoid any unintended consequences
	How will the outcomes of the campaign be evaluated to ensure the results are directly attributable to the campaign?	Pre- and post-campaign comparison of clinical surveillance data and changes in social cognition (e.g. knowledge, attitudes, behaviour, motivations)

### Step 4: Testing and implementing the SNAP-AMR Logic Model

**Fig 3:**
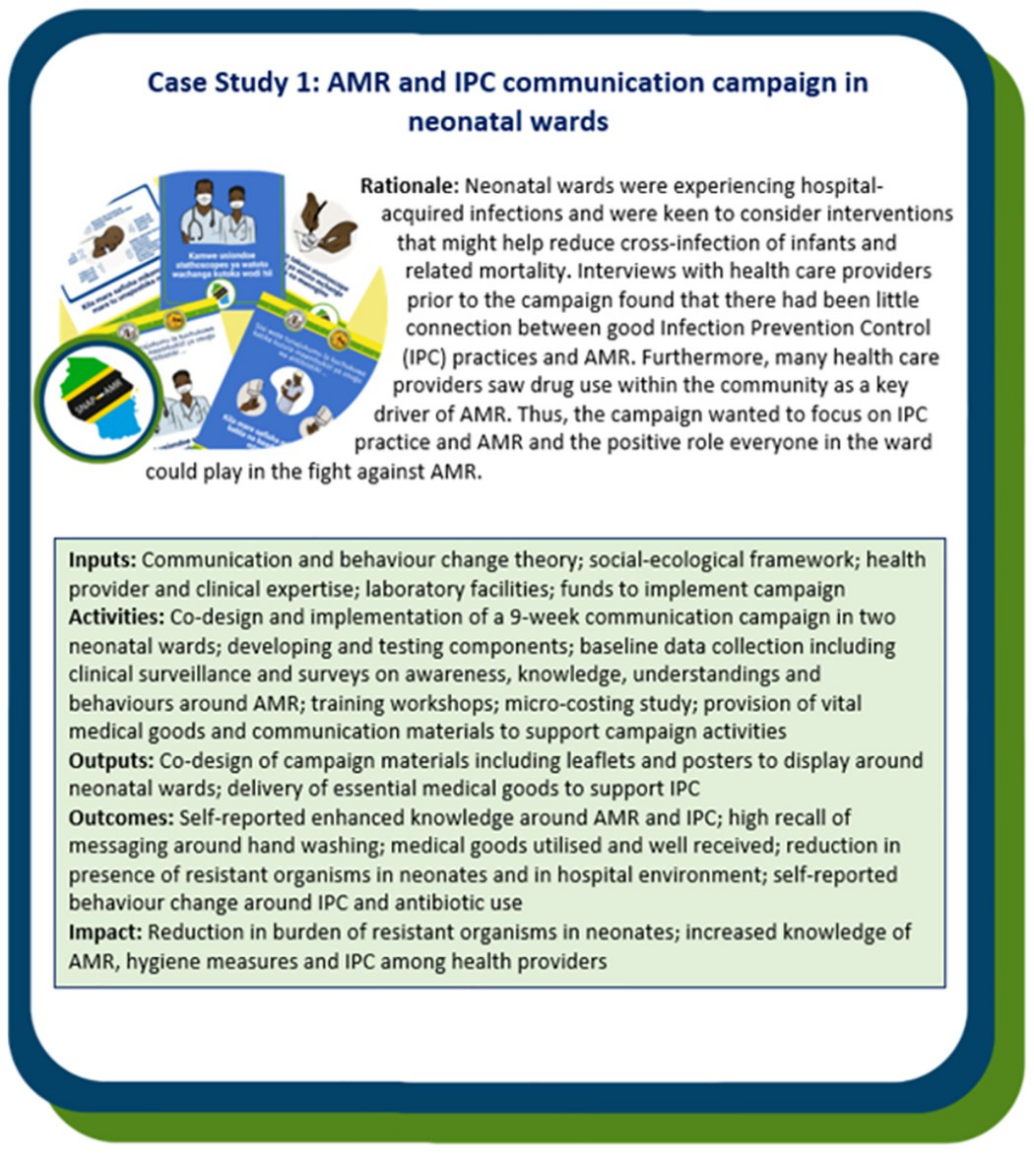
Case study 1—An AMR and Infection Prevention and Control (IPC) communication campaign conducted in two neonatal wards in northern Tanzania, at two zonal hospitals as part of the SNAP-AMR project.

**Fig 4:**
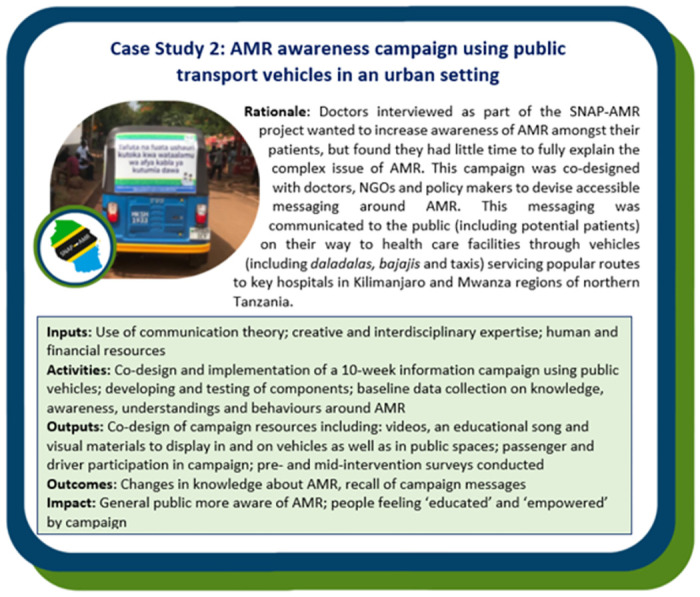
Case Study 2—A public-facing AMR awareness campaign conducted using vehicles servicing popular transport routes to two zonal hospitals in Kilimanjaro and Mwanza regions of northern Tanzania. See Laurie et al., (forthcoming) for full details on the planning, implementation, and evaluation of this campaign via co-design with healthcare providers in Tanzania.

**Fig 5:**
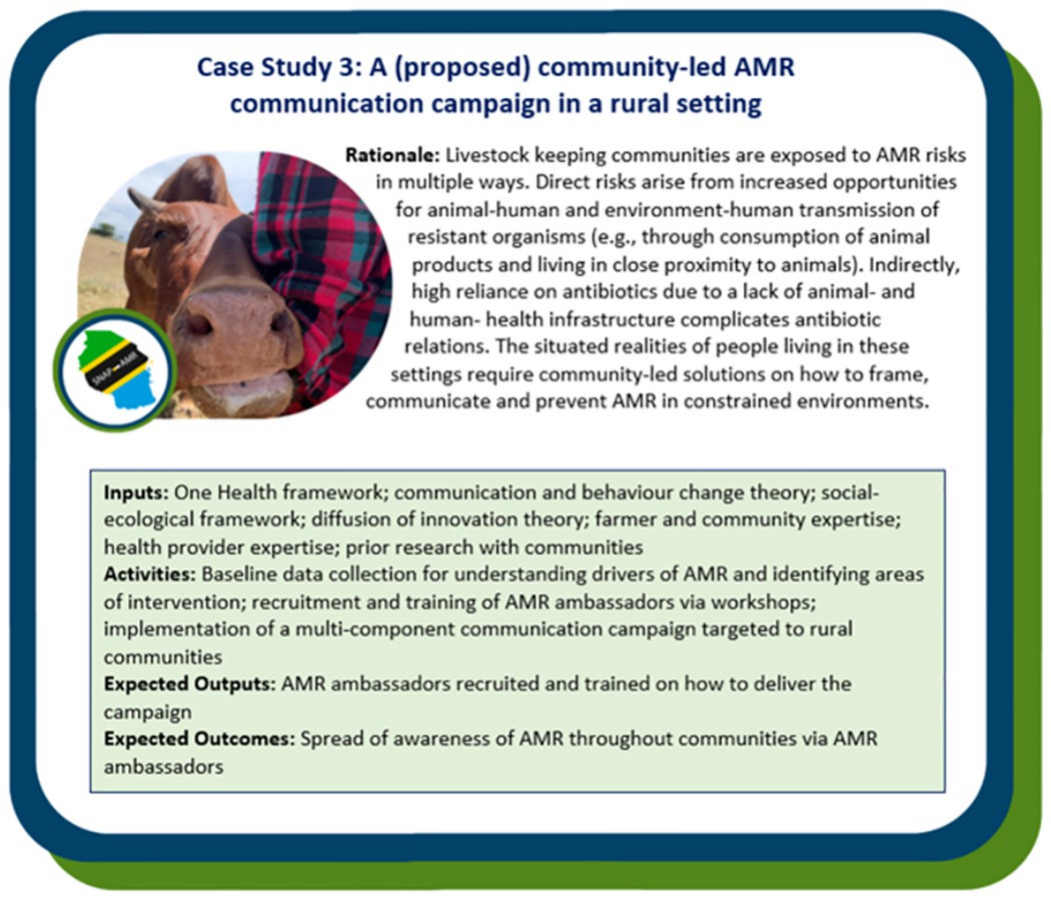
Case Study 3—A proposed community-led AMR communication campaign using local ambassadors in rural, livestock-dependent communities in northern Tanzania.

The Case Study boxes describe how the SNAP-AMR Logic Model was adapted to design AMR campaigns used across three different settings in northern Tanzania: a healthcare setting (Case Study 1, Fig 3), an urban setting (Case Study 2, Fig 4) and a rural setting (Case Study 3, Fig 5). The probing questions in Table 1 were used as a guide to inform each campaign.

As demonstrated in the Case Studies, each utilised the same SNAP-AMR Logic Model in Fig 2 to design a bespoke campaign carefully considering the mechanisms of action (i.e. inputs, activities) and how these map onto mechanisms of change (i.e. outputs, outcomes and impact) as appropriate for their specific context and target population(s). This highlights the utility of the SNAP-AMR Logic Model in providing the overarching conceptual campaign design, whilst considering the vital importance of context in ensuring the campaigns were place-based and locally specific.

## Discussion

This paper describes the process of developing the SNAP-AMR Logic Model for several interrelated AMR communication campaigns and activities in northern Tanzania. The model can be considered as the overarching conceptual base for structuring campaign activities and the framework for which links between inputs and activities can be mapped out onto outputs and outcomes. Below, we focus on the foundational stages of the SNAP-AMR Logic Model, namely the *Input Stage* and *Activities Stage* to highlight their critical role in delivering successful AMR campaigns.

## SNAP-AMR Logic Model Inputs Stage

The ultimate goal of all three campaigns was to reduce the burden of AMR in northern Tanzania, across health care, urban and rural settings. However, each individual campaign had its own aims and expected outcomes and, thus, had to carefully consider how inputs could be configured to maximise campaign activities. We describe how theory, frameworks and stakeholders were vital inputs for the SNAP-AMR Logic Model and cannot be overlooked.

### Theories used within the SNAP-AMR Logic Model

Utilising an overarching theoretical framework was essential for informing each of the three SNAP-AMR campaigns. Health campaigns that are theoretically underpinned in ways which take seriously the complexities of communicating around healthcare are considered more effective than those lacking a theoretical base [42]. Embedding campaigns within a chosen theoretical base provides a framework for understanding the likely process through which the campaign will achieve change and helps to identify and strengthen any gaps in the proposed activities.

Behavioural change and communication theories, as outlined in Box 1, were particularly relevant for the SNAP-AMR campaigns as it has been repeatedly shown that providers’ and publics’ behaviours are potential drivers of AMR, e.g., through inappropriate use of antimicrobials, poor Infection Prevention and Control (IPC) practices, shared animal and human environments, poor waste disposal and sub-optimal prescribing practices [43]. While we are acutely attuned to the structural drivers of such behaviours, we also recognise that publics and providers have the right to vital health knowledge to inform and empower them in decision making regarding their (patients) health. AMR awareness raising communication campaigns, embedded alongside wider policy and advocacy work, can therefore play a crucial role in efforts to reduce AMR where these campaigns are appropriate to individuals’ existing knowledge systems, and their role within antimicrobial stewardship (Ibid.) [43]. The behavioural change and communication theories used prompted consideration on how best to frame and target messages; how to segment audiences based on need; the type, length, appropriateness and exposure to various media information sources; and the role of social networks in determining how new information may travel in communities.

As shown in Case Studies 1 and 3, communication and behaviour change theories alone are not sufficient to bring about desired changes in practices around antibiotics. Studies have shown that health campaigns are most likely to be effective if they embrace a social-ecological perspective [36–37). That is, campaigns should consider the wider contextual factors that may influence health behaviour. Such an approach orients the focus away from the individual to the broader socio-ecological environment which may inhibit or facilitate health promoting behaviours. In relation to AMR, limited resources influence everything from the working and material conditions within health systems, to the testing and treatment available to publics and providers, to an individual’s ability to interpret and act on messages communicated [13]. For example, in *Case Study 1*: *AMR and IPC communication campaign in neonatal wards*, the involvement with health providers in co-design of the campaign highlighted the inhibiting structural and systemic barriers for optimal behaviours IPC behaviours including limited access to vital goods such as hand sanitiser and fresh gowns. The SNAP-AMR campaign, therefore, provided the required goods for providers and mothers to act on the accompanying messaging.

While the closed setting of the neonatal wards facilitated provisions of goods alongside messaging, we recognise that not all campaigns can include provision of goods. Nonetheless, it is fundamentally unethical to deny people information about AMR and associated optimal behaviours. Great care, however, is required to ensure that such messaging neither blames nor stigmatises individuals and their health behaviours. As we have demonstrated in *Case Study 2*: *AMR awareness campaign using public transport vehicles in urban settings*, it is possible to be cognisant of constraining environments and health seeking behaviours they elicit, while delivering campaigns which empower individuals by avoiding blame narratives and stigmatising messages (see Laurie et al., forthcoming). The campaigns also provide reasons for following suggested health behaviours grounded in the local context of everyday life and commonly understood examples. Such work can, and should, run alongside and not counter to advocating for more global systemic change.

To date, AMR awareness campaigns have predominantly focused on framing AMR as a human health issue. However, as shown in *Case Study 3*: *A (proposed) community-led AMR communication campaign in a rural setting*, there is a need to create awareness not only around human health but also animal health [44]. Consequently, we considered a One Health framing for the rural campaign setting. How humans and animals interact with their environment and how these interactions contribute to the overall AMR problem is poorly considered in AMR communication campaigns (cf. [8]). This is especially important in settings where humans and animals co-habit closely within their environment, such as amongst pastoral, agropastoral and smallholder communities within Tanzania and similar rural agricultural contexts in Africa and Asia. Integration of human and animal health is often a given in such settings, yet there are important links between human, animal and environmental spheres of life [45], with implications for spread and development of AMR. Such contexts constitute a dynamic and complex web of interaction whereby there are many paths by which drug residues and resistant bacteria can disseminate between humans, animals and the environment [44]. Here, a more integrated, One Health, approach is required, where AMR information is framed beyond human health, to target and include different sectors and a broader array of stakeholders from across society in campaign development and delivery. This does inevitably prolong the development of campaigns, for example as we experienced in Case Study 3, in adopting the One Health approach meant far longer had to be spent at the *Input* and *Activities* stage to fully comprehend the complexity of the issue, hence why this campaign development remains ongoing.

Framing AMR as a One Health issue would support efforts to reduce the AMR action gap by integrating the multiple spheres of life that AMR impacts (animal, human and environmental). This makes the issue more relevant to the broader communities affected (patients, caretakers, livestock keepers). It further expands the scope of responsibility by moving the focus to societal-wide change which takes account of the local barriers and constraints when developing individualistic campaign messages. This helps expand the size of the population who consider AMR to have relevance to their lives, increasing the number of people and organisations who may take action to address it.

### Stakeholders/local champions

Considering the role of stakeholders and local ambassadors was vital across all campaigns. Working collaboratively with members from the community has been shown to be critical to the successful control of infectious diseases in sub-Saharan Africa [46]. Campaigns which incorporate co-design and community participation are known to help build empowerment and community resilience to health challenges and are especially effective in tackling health inequalities [47]. For example, in *Case Study 3*: *A (proposed) community-led AMR communication campaign in rural setting*, initial work within the *Input* stage highlighted that sources of health information spread through social networks (see [13]), with the expertise of someone’s health knowledge frequently related to trusted status rather than formal health training. This indicates that dedicated AMR champions nominated by community peers themselves are likely to play a central role in community campaigns. Likewise, in *Case Study 1*: *AMR and IPC communication campaign in neonatal wards*, work at the *Input* stage revealed the importance of using known doctors within health settings to deliver vital training and messaging.

### SNAP-AMR logic model activities stage

Co-design, targeted messaging, utilising a multi-pronged approach and utilising/generating an evidence base were central tenets of all three campaign activities within the project.

#### Co-design

Co-design is a core guiding principle within SNAP-AMR, with regards to project research, the design of the SNAP-AMR Logic Model and the individual campaigns. Working in a collaborative and inclusive manner ensured that the SNAP-AMR Logic Model *and* individual campaigns were always aligned with the principles of the Tanzanian NAP, and was sensitive to the needs, contexts and priorities of in-country health systems and communities. In addition, co-design builds and fosters long-term relationships by frequently bringing stakeholders and project members together around shared goals and visions, while respecting and valuing the knowledge each stakeholder has. As well as supporting buy-in from key change makers, co-design also promotes longer-term sustainability beyond the project lifespan by ensuring the campaigns are funded but, crucially, not owned by a project. Furthermore, in valuing the contextual knowledge of diverse stakeholders, co-design ensures the project avoids any unintended consequences within the target audience. For example, in *Case Study 1*: *AMR and IPC communication campaign in neonatal wards*, the health care providers workshop on messaging revealed that messages commonly used within HICs relating to decreased drug efficacy could lead to their patients avoiding vital medication and undo hard-fought gains to encourage publics to engage with health services. Similarly, in *Case Study 2*: *An AMR awareness campaign using public transport vehicles in an urban setting* we conducted several workshops with health providers, policymakers and vehicles drivers to ensure campaign messages, materials and implementation strategies were locally specific and contextually appropriate.

#### Targeted messaging

Adopting a co-design approach ensured that the messaging of the SNAP-AMR campaigns was targeted to distinct audiences and considered the place-specific contexts. Messages should be informed by the evidence base around key drivers of AMR specific to the local context in order to mitigate unintended consequences, improve uptake of recommended preventative actions and improve understanding of recommendations. Yet, many AMR awareness campaigns focus on blanket messaging targeted at individuals about antimicrobial consumption [8]. Campaigns with a focus on overuse/antibiotic use might be appropriate in a HRS, however, antibiotic use may not be the main driver of AMR in other settings. For example, in Tanzania high prevalence of antibiotic resistant bacteria in livestock-owning communities is associated with local practices affecting transmission such as access to water sources shared with livestock and consumption of milk [48]. The latter could act as a source of antibiotic-resistant bacteria or antibiotic residues either directly or via contaminated milk-storage vessels. Therefore, in this context, as part of the proposed *Case Study 3*: *Case Study 3*: *A (proposed) community-led AMR communication campaign in rural setting* specific messages on strategies to reduce transmission of infection by targeting these drivers will be a central focus.

Similarly, in *Case Study 1*: *AMR and IPC communication campaign in neonatal wards* messages on AMR included those under a unifying banner of ‘we all have a role to play’ followed by targeted messaging for specific groups including doctors, nurses, ward attendants and mothers. Care was taken to emphasise that everyone can play a role but also specifically phrase this in a positive, empowering and action-oriented way in order to not scare or intimidate audiences, which could have discouraged people engaging with the campaign.

#### Multi-pronged approach

All three campaigns leveraged multiple platforms and modes of delivery in order to deliver campaign messages. Multimodal mass media campaigns have demonstrated an effect on the amount of antibiotics used in Europe and the UK [49], whereas campaigns that rely on materials such as posters and leaflets as standalone resources were not effective in leading to behaviour change [50–53]. A literature review of behaviour change and antibiotic prescribing found that media campaigns are more effective than medical professionals in disseminating information about antibiotics, but medical professionals are more effective at influencing behaviour change over media campaigns [54]. The review was conducted within Europe, and little has been found to understand the roles of social networks of influence and trusted sources of knowledge within countries such as Tanzania. Accordingly, we ensured the entirety of the SNAP-AMR Logic Model was underpinned with an understanding of context (i.e. through consideration of the social-ecological framework). This was vital in all SNAP-AMR Campaigns, especially *Case Study 2*: *AMR awareness campaign using public transport vehicles in urban settings*, which aimed to garner the attention of passengers and so employed external visuals, internal posters, bespoke AMR song written by Nicholas Materu, flyers and drivers as vital conduits of information.

This study has some limitations. While the campaigns were evaluated through formative and summative assessments to test feasibility and assess impacts of the campaign, conducting a process evaluation may have been beneficial to assess whether the campaigns components were being implemented as intended. Furthermore, the process of developing the logic model was interrupted by COVID-19 which impacted the extent of co-design we could achieve. Our desire to develop multiple iterations of the model was impacted due to reduced time commitments from team members and stakeholders to dedicate to design of the model. Additionally, given that one of the campaigns was aimed at the general public, the campaign could have benefitted from stakeholders beyond researchers and medical professionals who have expertise in designing public campaigns.

## Conclusion

Designing effective communication campaigns that are impactful, evidence-based and contextually appropriate is essential for addressing the global health threat presented by AMR. This paper outlines the process of creating the SNAP-AMR Logic Model, which serves as a comprehensive framework for multiple interconnected AMR communication campaigns and activities in northern Tanzania. The SNAP-AMR Logic Model effectively encompasses all the necessary elements for designing, implementing and evaluating AMR communication interventions across various healthcare settings, urban areas and rural communities in northern Tanzania.

We highlight the value of logic models as indispensable tools for government bodies and advocacy stakeholders to implement campaigns tailored to their specific contexts. We anticipate that this work will provide significant contributions to the newly established technical working group (TWG) for Monitoring and Evaluation, as well as the existing Awareness and Education TWG within the Tanzania National Action Plan on AMR (2023–2028). Although this framework is presented within the context of Tanzania, it has broader applicability in the development of campaigns addressing various health communication issues across low-, middle- and high-income countries.

## Supporting information

S1 Checklist (DOCX)
